# Supplementary material for: Reporting and interpreting non-significant results in animal cognition research
Source: PeerJ. 2023 Mar 9;11:e14963. doi: 10.7717/peerj.14963 (PMC10008313; doi:10.7717/peerj.14963)
Supplement: Supplemental Information 1 [file peerj-11-14963-s001.docx]

**Supplemental Material to:**

**Guidance – remove this box before submitting!**

Yellow callout boxes provide general notes. Please remove the yellow boxes before submitting. For full guidance see <https://peerj.com/about/author-instructions>

Blue highlighted example text should be replaced or removed with your own information.

| **DO**  –Use clear and grammatically correct English.  –Save as US Letter size format.  –Ensure line numbering is enabled.  –Align text LEFT.  –Ensure title, abstract, and author information matches what is entered online during submission. | **DO NOT**  –Embed ANY figures or tables in the text. Instead, upload a separate file for each on the file uploads page when submitting. Example – If you have 3 figures, then you will upload 3 figure files & be asked to add a figure title for each. See <https://peerj.com/about/author-instructions/#figures> for figure formats. |
| --- | --- |

**Reporting and interpreting non-significant results in animal cognition research**

Benjamin G. Farrar^1, 2^, Alizée Vernouillet^3^, Elias Garcia-Pelegrin^1,4^, Edward W. Legg^5, 6, 7^, Katharina F. Brecht^8^, Poppy Lambert^9^, Mahmoud Elsherif^10,11^ Shannon Francis^11^, Laurie O’Neill^12^, Nicola S. Clayton^1^, Ljerka Ostojić^5, 6, 7^

^1^Department of Psychology, University of Cambridge, UK

^2^Institute for Globally Distributed Open Research and Education (IGDORE), UK

^3^Department of Experimental Psychology, Universiteit Gent, Belgium

^4^Department of Psychology, National University of Singapore, Singapore

^5^Department of Psychology, Faculty of Humanities and Social Sciences, University of Rijeka, Croatia

^6^Division of Cognitive Sciences, Faculty of Humanities and Social Sciences, University of Rijeka

^7^Centre for Mind and Behaviour, University of Rijeka, Croatia

^8^Institute for Neurobiology, University of Tübingen, Germany

^9^Messerli Research Institute, University of Veterinary Medicine Vienna, Austria

^10^Department of Psychology, University of Birmingham, UK

^11^Department of Neuroscience, Psychology and Behaviour. University of Leicester, UK

^12^Comparative Cognition Research Group, Max Planck Institute for Ornithology, Germany

Corresponding Authors:

Benjamin G. Farrar

20 Bosworth Road

Cambridge

CB1 8RG

United Kingdom

Email address: [farrarbg@gmail.com](mailto:farrarbg@gmail.com)

Ljerka Ostojić

Department of Psychology

Faculty of Humanities and Social Sciences

Sveučilišna avenija 4

51000 Rijeka

Croatia

Email address: [lj.ostojic@uniri.hr](mailto:lj.ostojic@uniri.hr)

**Results of the Reliability and Quality Control coding**

*Double-Blind Coding*

Only five of the 24 article titles were identified as containing statements referring to statistically non-significant results by either of the two coders, and from this, the two coders agreed on only one out of five (20%) of the articles about whether the title included a statement that reported or interpreted a statistically non-significant result. Following discussion with the whole group, we agreed that it was often ambiguous whether the titles of articles were reporting the results or already interpreting them in relation to a substantive claim, and so we decided to combine these variables and have no sub-group analysis for the title claim, deviating from our original plan. When considering the category (Justified; Caveated, Similar or Ambiguous; No Effect) of the title claim, the two coders agreed on two out of six articles (33%; Gwet’s AC1 = 0.16, 95% CI: -0.60 to 0.92, *p* = .615; Cohen’s kappa = 0.07, *p* = .674)). Three of the four disagreements occurred when one coder did not interpret the title as referring to statistically non-significant result, e.g. as in “Evidence that novel flavors unconditionally suppress weight gain in the absence of flavor-calorie associations” (Seitz et al., 2020), and one where a coder appeared to have made an error. From discussion within the group, it was evident that these ambiguous cases — where the statements were not clearly written referring to statistically non-significant results but involved an interpretation that did not directly corresponds to a specific non-significant result from the article — proved the most difficult during the whole coding procedure, and this affected the reliability of the title claims and population claims from the abstract.

The coders identified 24 statements from the abstracts of the papers that reported a statistically non-significant result, from which they coded the same claim on 22 out of 24 occasions (91.6%). Of these 22 claims, the two coders agreed on 19 of their classification (86.3%; Gwet’s AC1 = 0.83, 95% CI: 0.62 to 1.00, *p* < .001; Cohen’s kappa = 0.68, *p* < .001). In contrast, the coders identified only eight statements that interpreted a statistically non-significant result in relation to a substantive claim from the abstracts of articles, from which they agreed on three occasions (37.5%), and of these three, agreed on two of their classifications (66.7%; Gwet’s AC1 = 0.61, 95% CI: -1.27 to 1.00, *p* = 0.296). From the results, the coders recorded the same text for 15 of the 22 (68.2%) abstract claims that they had coded the same, and of these 15, they agreed on 13 of their classification (86.7%; Gwet’s AC1 = 0.82, 95% CI: 0.55 to 1.00, *p* < .001; Cohen’s kappa = 0.75, *p* = .001) and extracted the exact same *p*-value for 10 of these 13 (76.9%).

In sum, the double-blind coding demonstrated good inter-rater consistency for how the abstract reported non-significant results and associated results and *p*-values were extracted, even before our quality control procedures had been implemented, which ensured that any potential errors during coding are rectified before data analysis. In contrast, inter-rater consistency was low for the title claims and interpretations of the results from the abstracts. This matched our subjective experience of the coding procedure, where we experienced many cases where the interpretation of the results was vague and about a theoretical hypothesis that did not closely correspond to any specific statistical result from the article. In contrast, the statement reporting a non-significant result in the abstract could often be easily mapped onto a particular statistically non-significant result in the text.

*Quality Control*

To rectify any potential errors during coding, each article was checked by a quality controller. The initial coders identified 67 possible statements referring to a statistically non-significant result in the titles of papers, and the quality controller agreed with the classification of 39 (58%) of these statements, had a minor disagreement with six statements (9%), and a major disagreement with 22 statements (33%). Of note, 16 of these 22 major disagreements came from a single repeated error in which one individual coder coded ‘ambiguous’ for titles containing no statement referring to a statistically non-significant result. In the abstract, coders identified 281 statements reporting a statistically non-significant result in the paper. Of these, the quality controllers agreed with the classification of 250 (89%), had minor comments about 16 (6%), and major disagreements with 15 (5%). Coders identified a much smaller number of interpretations of statistically non-significant results in relation to a substantive claim in the articles and disagreed more frequently: Of the 82 identified statements, the quality controllers agreed with the classification of 44 (53%), had minor comments about 18 (22%) and major disagreements with 20 (24%). Regarding the result texts from the article bodies, coders identified 282 results, of which the quality controller agreed with the classification and extracted *p*-value for 252 (89%), had minor comments for 13 (5%), and major disagreements for 17 (6%).

In addition to its primary aim, i.e., to identify any clear errors in the data extraction process, this quality control process allowed us to also to i) highlight borderline cases where our coding scheme could not clearly categorize certain statements, and ii) assess the robustness of the coding procedure. In line with the results from the double-blind coding, the quality control process demonstrated a high inter-rater agreement and consistency with identifying and classifying statements reporting statistically non-significant results from abstracts, and the corresponding results and *p*-values from the main text, yet greater inconsistency in deciding, i) whether titles and interpretations of the results in relation to a substantive claim really pertained to the result of a non-significant NHST, and ii) whether the authors were claiming the absence of an effect from these statistically non-significant results. This inconsistency occurred mainly because many titles and interpretations of results in relation to a substantive claim referred not to a certain statistical result but made a *vague* theoretical statement.

**Prisma Checklist**

| **Section and Topic** | **Item #** | **Checklist item** | **Location where item is reported** |
| --- | --- | --- | --- |
| **TITLE** | | |  |
| Title | 1 | Identify the report as a systematic review. | Title |
| **ABSTRACT** | | |  |
| Abstract | 2 | See the PRISMA 2020 for Abstracts checklist. | Complete for all non-NA items below |
| **INTRODUCTION** | | |  |
| Rationale | 3 | Describe the rationale for the review in the context of existing knowledge. | Introduction |
| Objectives | 4 | Provide an explicit statement of the objective(s) or question(s) the review addresses. | Introduction, lines 133-139 |
| **METHODS** | | |  |
| Eligibility criteria | 5 | Specify the inclusion and exclusion criteria for the review and how studies were grouped for the syntheses. | Methods – data extraction and classification section |
| Information sources | 6 | Specify all databases, registers, websites, organisations, reference lists and other sources searched or consulted to identify studies. Specify the date when each source was last searched or consulted. | Methods – lines 144-151 |
| Search strategy | 7 | Present the full search strategies for all databases, registers and websites, including any filters and limits used. | NA – a hand search of specific journals was performed |
| Selection process | 8 | Specify the methods used to decide whether a study met the inclusion criteria of the review, including how many reviewers screened each record and each report retrieved, whether they worked independently, and if applicable, details of automation tools used in the process. | Methods – data extraction and classification section |
| Data collection process | 9 | Specify the methods used to collect data from reports, including how many reviewers collected data from each report, whether they worked independently, any processes for obtaining or confirming data from study investigators, and if applicable, details of automation tools used in the process. | Methods – data extraction and classification section |
| Data items | 10a | List and define all outcomes for which data were sought. Specify whether all results that were compatible with each outcome domain in each study were sought (e.g. for all measures, time points, analyses), and if not, the methods used to decide which results to collect. | Methods - – data extraction and classification section and Table 2 and Table 3 |
|  | 10b | List and define all other variables for which data were sought (e.g. participant and intervention characteristics, funding sources). Describe any assumptions made about any missing or unclear information. | As above |
| Study risk of bias assessment | 11 | Specify the methods used to assess risk of bias in the included studies, including details of the tool(s) used, how many reviewers assessed each study and whether they worked independently, and if applicable, details of automation tools used in the process. | NA – not relevant to review objective |
| Effect measures | 12 | Specify for each outcome the effect measure(s) (e.g. risk ratio, mean difference) used in the synthesis or presentation of results. | Methods – data extraction and classification section. The review goal was descriptive. |
| Synthesis methods | 13a | Describe the processes used to decide which studies were eligible for each synthesis (e.g. tabulating the study intervention characteristics and comparing against the planned groups for each synthesis (item #5)). | Methods – analysis section, lines 231 - 246 |
|  | 13b | Describe any methods required to prepare the data for presentation or synthesis, such as handling of missing summary statistics, or data conversions. | NA |
|  | 13c | Describe any methods used to tabulate or visually display results of individual studies and syntheses. | NA, only aggregate data with examples were presented |
|  | 13d | Describe any methods used to synthesize results and provide a rationale for the choice(s). If meta-analysis was performed, describe the model(s), method(s) to identify the presence and extent of statistical heterogeneity, and software package(s) used. | NA |
|  | 13e | Describe any methods used to explore possible causes of heterogeneity among study results (e.g. subgroup analysis, meta-regression). | NA |
|  | 13f | Describe any sensitivity analyses conducted to assess robustness of the synthesized results. | NA |
| Reporting bias assessment | 14 | Describe any methods used to assess risk of bias due to missing results in a synthesis (arising from reporting biases). | NA |
| Certainty assessment | 15 | Describe any methods used to assess certainty (or confidence) in the body of evidence for an outcome. | NA |
| **RESULTS** | | |  |
| Study selection | 16a | Describe the results of the search and selection process, from the number of records identified in the search to the number of studies included in the review, ideally using a flow diagram. | Results – lines 249 – 254 and PRISMA diagram (Figure 1) |
|  | 16b | Cite studies that might appear to meet the inclusion criteria, but which were excluded, and explain why they were excluded. | PRISMA diagram (Figure 1) |
| Study characteristics | 17 | Cite each included study and present its characteristics. | Available in open dataset |
| Risk of bias in studies | 18 | Present assessments of risk of bias for each included study. | NA |
| Results of individual studies | 19 | For all outcomes, present, for each study: (a) summary statistics for each group (where appropriate) and (b) an effect estimate and its precision (e.g. confidence/credible interval), ideally using structured tables or plots. | NA |
| Results of syntheses | 20a | For each synthesis, briefly summarise the characteristics and risk of bias among contributing studies. | NA |
|  | 20b | Present results of all statistical syntheses conducted. If meta-analysis was done, present for each the summary estimate and its precision (e.g. confidence/credible interval) and measures of statistical heterogeneity. If comparing groups, describe the direction of the effect. | NA |
|  | 20c | Present results of all investigations of possible causes of heterogeneity among study results. | Heterogeneity within the descriptive results forms a central part of the discussion. |
|  | 20d | Present results of all sensitivity analyses conducted to assess the robustness of the synthesized results. | NA |
| Reporting biases | 21 | Present assessments of risk of bias due to missing results (arising from reporting biases) for each synthesis assessed. | NA |
| Certainty of evidence | 22 | Present assessments of certainty (or confidence) in the body of evidence for each outcome assessed. | NA |
| **DISCUSSION** | | |  |
| Discussion | 23a | Provide a general interpretation of the results in the context of other evidence. | Discussion - Lines 317-327 |
|  | 23b | Discuss any limitations of the evidence included in the review. | Discussion – Lines 360 to 366, and Results, throughout the inter-rater reliability sections |
|  | 23c | Discuss any limitations of the review processes used. | Discussion – Lines 360 to 366, and Results, throughout the inter-rater reliability sections |
|  | 23d | Discuss implications of the results for practice, policy, and future research. | Discussion – lines 328 to 359 |
| **OTHER INFORMATION** | | |  |
| Registration and protocol | 24a | Provide registration information for the review, including register name and registration number, or state that the review was not registered. | Details of the preregistration are presented in lines 144-145 |
|  | 24b | Indicate where the review protocol can be accessed, or state that a protocol was not prepared. | A link to the protocol is provided in the methods |
|  | 24c | Describe and explain any amendments to information provided at registration or in the protocol. | Deviations from the original pre-registration and protocol and outlined and justified throughout the methods and results |
| Support | 25 | Describe sources of financial or non-financial support for the review, and the role of the funders or sponsors in the review. | Included in PeerJ submission |
| Competing interests | 26 | Declare any competing interests of review authors. | Included in PeerJ submission |
| Availability of data, code and other materials | 27 | Report which of the following are publicly available and where they can be found: template data collection forms; data extracted from included studies; data used for all analyses; analytic code; any other materials used in the review. | Methods |

*From:*  Page MJ, McKenzie JE, Bossuyt PM, Boutron I, Hoffmann TC, Mulrow CD, et al. The PRISMA 2020 statement: an updated guideline for reporting systematic reviews. BMJ 2021;372:n71. doi: 10.1136/bmj.n71

For more information, visit: <http://www.prisma-statement.org/>
